# Everybody's Page

**Published:** 1892-04-02

**Authors:** 


### Everybody's Page.

Chicago Scotchmen are going to build a hospital in that city. Needless to add it will be called the Burns' Free Hospital.

It appears that the wood anemone possesses properties which have an extremely energetic action on the human system. A French physician is reported to have extracted from it a substance in the form of crystalline needles, which in small doses has beneficial action in catarrh and chronic bronchitis or convulsive cough. But the drug is evidently one to be used with great caution, as in large doses it will produce paralysis.

Notwithstanding that Buxton, from its inland position and elevation, is naturally colder than our southern watering-places, which are also probably blessed with more sunshine, much is to be said in its favour as a winter resort for invalids in the early stage of consumption. It is asserted, and we believe correctly, that the colder the season there the greater is the proportion of recoveries, and the more marked the improvement in the health of the visitors.

A suggestion, which is certainly humane, has been put forward, that old paupers, who have behaved themselves with becoming propriety when in the workhouse, should after a period of probation be allowed to wear ordinary clothing. Seeing that a distinction is made in the case of the treatment of misdemeanants, it seems only logical that some distinction should be drawn between paupers who have come to grief through sheer misfortune, and those who have never done all they could for themselves and do not behave as they ought when kept by the State.

"Then snap your fingers at them, the germs of course we mean, and take to heart the warning of the Browns of Walham Green," so sings Geo. R. Sims, and really, although it sounds unscientific, it is advice most conducive to the peace of mind of the nervous individual. The "Annales de Micro-graphie" says that Dr. Freudenreich's experiments relate the fact that the cholera bacillus if put into milk just fresh drawn from the cow dies in about an hour, while goats' milk takes five hours to accomplish this. Typhoid bacillus takes twenty-four hours to die in cows' milk and five in goats' milk. Then comes the perplexing fact that Dr. Freudenreich finds that milk maintained at a temperature of 131 degrees Fah. for an hour loses its germicidal properties. The older the milk gets the less its destroying power becomes, and in four days it ceases to have any effect, and yet we boil our milk to render it pure.

An attack on a somewhat large scale is being made in America against cigarette smoking. Sweeping statements are made as to the deadly effect of this habit so dear to the youth of that go-ahead country, a habit which Congress is now being urged to suppress. An example in this direction has been set by the legislature of Ontaria, where the crusade evidently meets with favour. A Bill introduced by the Premier is stated to have been read a second time, which prohibits anyone under the age of 18 from smoking cigarettes, under the penalty of a fine not exceeding five dollars, while those who pander to youthful cravers after the seductive soother, are to be liable to a fine not exceeding fifty dollars. According to American papers, numerous deaths of *young men* under the age of 16 are directly traceable to the habit of smoking cigarettes. We always thought anyone of the male sex under the age of 16 was a boy, but in this as in other things on the other side of the Atlantic, they are far ahead of us worn-out folk.

Few things show more clearly the growth of the popularity of insurance in this country than the great expansion which has taken place in this business during the past half-century. Scarcely anything exists which cannot be insured, but anyone would have been smiled at as an irredeemable joker had he prophesied fifty years ago that insurance "against twins" would fall within the bounds of "practical business." That it has done so is evident from a circular recently issued from "Lloyd's."

The kindly care of the "National Anti-Tobacco Society" will soon be forthcoming to the "slaves of the pipe," and at a meeting at the Memorial Hall, Farringdon Street, the opponents of the worship of the goddess Nicotine came to all sorts of terrible conclusions about the harmfulness of tobacco. Students, we are told, have been noticed to decline in intelligence as they develop in tobacco-smoking, and yet a physiologist has just told us in our columns that tobacco-smoke enables us to play our parts in the social duties of conversation, assisted by a clear and active brain! We agree to differ; but, verily, moderation in all things is not one of the Britisher's chief attributes.

Recent investigations on cancer as an infective disease have pointed to some interesting and curious facts. It is, perhaps, superfluous to state that our old friend, or rather enemy, the microbe, who seems ever present in one shape or another, comes in for a share of abuse as having a good deal to do with the disease. What is more interesting to note is, that cancer is especially prevalent along the courses of rivers which overflow their banks. The transplanting experiments which have been made have been successful from dog to dog, but have produced nothing but negative results when the transplanting has been from men to animals.

Dr. Walter Tyrrell writes a strong article in the last quarter's *Bristol Medico-Chirurgical Journal* on "Malvern as a Health Resort," for those whose lungs or nervous system are in need of building up. The dryness of the air, the dryness of soil, the large amount of sunshine, and the equable nature of the temperature are no mean qualifications for a health resort. Year by year the numbers increase of invalids who fly to the sunny Riviera or the heights of Davos-Platz or St. Moritz, even of those whose pockets will not permit them the luxuries that make Continental life home-like. There is magic in the name of a foreign country, and yet we doubt whether the invigorating air of some of our English hills, coupled with their easy accessibility, weighed with the long Continental journey, is not oftentimes undervalued by medical men and their patients alike.

Combination seems to be the order of the day in every trade and profession. The British public will scarcely be surprised if the inmates of casual wards, some of whom have recently shown signs of dissatisfaction with their treatment, follow the example set them and strike for down quilts an Havannah cigars of the finest brands. One of these gentlemen, who earn the gratitude of their country by their usefulness in general, was much incensed a few days ago at finding the ward into which he applied for admission was full. His wrath is accounted for by the fact that he so timed his arrival, as he hoped, as to reach the ward at supper time. Unfortunately, his watch must have been slow, for there was no supper for him, and he could not gain admission till midnight. This was not the end of his ill-treatment, f r he objected to the manner in which the ward was warmed. It seems monstrous that this deserving profession should not receive every luxury that civilisation can produce.

